# Draft Genome Sequence of *Salmonella enterica* subsp. *enterica* Serovar Mishmarhaemek Isolated from Bovine Feces

**DOI:** 10.1128/genomeA.01210-15

**Published:** 2015-10-15

**Authors:** Ashley Cooper, Dominic Lambert, Adam G. Koziol, Karine Seyer, Catherine D. Carrillo

**Affiliations:** a Canadian Food Inspection Agency, Government of Canada, Ottawa, Canada; b Canadian Food Inspection Agency, Government of Canada, Saint-Hyacinthe, Canada

## Abstract

*Salmonella enterica* subsp. *enterica* serovar Mishmarhaemek is a Gram-negative, non-spore-forming, rod-shaped bacterium implicated in human clinical disease. Here, we report a 4.8-Mbp draft genome sequence of a nalidixic acid-resistant isolate of *S.* serovar Mishmarhaemek.

## GENOME ANNOUNCEMENT

*Salmonella enterica* subsp. *enterica* serovar Mishmarhaemek was first isolated from an infected patient who developed symptoms of acute enteritis following consumption of cream pastries in Mishmar-Haemek, Israel (1). This bacterium belongs to antigenic group O:13 (formerly G_1_-G_2_), along with *S*. serovar Cubana and *S*. serovar Poona, and contains somatic O antigens 1,13,23, and flagellar H antigens d (phase 1) and 1,5 (phase 2) (1,13,23:d: 1,5) (2). There are very few reports of *S*. serovar Mishmarhaemek in the literature. A study conducted in 2003 of *Salmonella* isolated from slaughtered cattle in Ethiopia found 48% of positive sample isolates to be *S*. serovar Mishmarhaemek (3). One of these isolates, obtained by the Canadian Food Inspection Agency (designated OLC-1602) has the distinguishing feature of being naturally resistant to nalidixic acid.

Genomic DNA was extracted from overnight cultures grown on brain heart infusion (BHI) agar using the Promega, Maxwell 16 Cell DNA purification kit (Promega, Madison, WI). Sequencing libraries were constructed using the Nextera XT DNA sample preparation kit and paired-end sequencing was performed on the Illumina MiSeq platform (Illumina, Inc., San Diego, CA), using a 500-cycle MiSeq reagent kit (version 2). A total of 1,439,739 paired-end reads were generated. Sequencing errors in reads were corrected using Quake version 0.3 with a k-mer size of 15 (4) and assembled *de novo* using SPAdes v. 3.1.1 (5). Assembly resulted in 43 contigs greater than 1,000 bp, with an average 44-fold genome coverage. The combined length of the contigs is 4.8 Mbp with a G+C content of 52%. Gene annotations were performed with the National Center for Biotechnology Information (NCBI) Prokaryotic Genome Annotation Pipeline (PGAP) (6), which predicted 4,515 coding sequences (CDS) and 116 RNA genes.

To our knowledge, no other genomic data for *S*. serovar Mishmarhaemek exists in the literature. Analysis of the *gyrA* gene, associated with nalidixic acid resistance (Nal^R^), found a single point mutation located in the codon at amino acid 87-Asp. This mutation resulted in an 87-GAC (Asp) → GCC (Ala) mutation. Point mutations often associated with Nal^R^ in *Salmonella* are Asp-87 substitutions to Asn, Gly, or Tyr (7, 8). Further analysis of the *S*. serovar Mishmarhaemek *gyrA* coding region resulted in a single match to a ciprofloxacin-resistant *S*. serovar Typhi isolated in Kuwait which contained the same uncommon amino acid substitution (8).

### Nucleotide sequence accession numbers.

This whole-genome shotgun project has been deposited at DDBJ/EMBL/GenBank under the accession number JZUU00000000. The version described in this paper is the first version, JZUU01000000.
